# *In vitro* activity of SPR719 against *Mycobacterium ulcerans*, *Mycobacterium marinum* and *Mycobacterium chimaera*

**DOI:** 10.1371/journal.pntd.0009636

**Published:** 2021-07-26

**Authors:** Sacha J. Pidot, Jessica L. Porter, Troy Lister, Timothy P. Stinear

**Affiliations:** 1 Department of Microbiology and Immunology at the Doherty Institute, University of Melbourne, Melbourne, Australia; 2 Spero Therapeutics, Cambridge, Massachusetts, United States of America; Stanford University, UNITED STATES

## Abstract

Nontuberculosis mycobacterial (NTM) infections are increasing in prevalence across the world. In many cases, treatment options for these infections are limited. However, there has been progress in recent years in the development of new antimycobacterial drugs. Here, we investigate the *in vitro* activity of SPR719, a novel aminobenzimidazole antibiotic and the active form of the clinical-stage compound, SPR720, against several isolates of *Mycobacterium ulcerans*, *Mycobacterium marinum* and *Mycobacterium chimaera*. We show that SPR719 is active against these NTM species with a MIC range of 0.125–4 μg/ml and that this compares favorably with the commonly utilized antimycobacterial antibiotics, rifampicin and clarithromycin. Our findings suggest that SPR720 should be further evaluated for the treatment of NTM infections.

## Author summary

Nontuberculosis mycobacteria represent a large group of diverse bacteria that live across a range of environments. Human contact with the habitats in which these organisms live can result in opportunistic infections. Among the NTM, *Mycobacterium ulcerans* causes necrotic skin ulcers that can lead to significant long term physical impairment; *Mycobacterium marinum* causes granulomatous skin lesions; and *Mycobacterium chimaera* has been linked to contaminated heater-cooler units used during cardiac surgery, resulting in prosthetic heart valve infections that are particularly difficult to treat. We performed laboratory experiments to test the susceptibility of these NTM species (*M*. *ulcerans*, *M*. *marinum* and *M*. *chimaera*) to a recently developed antibiotic, SPR719. We found that SPR719 inhibits the growth of these mycobacteria at concentration ranges similar to or better than commonly used anti-mycobacterial antibiotics. As SPR720, the oral prodrug of SPR719, has recently completed a Phase I safety, tolerability and PK study in healthy human volunteers, the potential exists for this product to be explored for the treatment of NTM infections, where new treatment options are urgently needed.

## Introduction

Non-tuberculosis mycobacteria (NTM) is a catch-all descriptor for mycobacteria other than *Mycobacterium tuberculosis* and *Mycobacterium leprae*, the causative agents of tuberculosis and leprosy, respectively. NTM can theoretically be any of the more than 170 named species within the genus mycobacteria. NTM are distributed widely across different environments, are often isolated from soil and water and some are considered opportunistic pathogens. NTM are also often antibiotic and disinfectant resistant, complicating treatment options [1]. While not all NTM are capable of causing human infections, several species including *Mycobacterium abscessus*, *Mycobacterium avium*, *Mycobacterium ulcerans*, *Mycobacterium marinum*, and *Mycobacterium chimaera* can cause a range of infections in different organs and at different anatomical sites, including the lungs, skin, subcutaneous tissue and cardiac associated prosthetic medical devices.

*M*. *ulcerans* causes the neglected tropical skin disease known as Buruli ulcer (BU) [2]. The disease is endemic in several regions of Africa, primarily in poor and rural communities [3]. First presenting as a small nodule, if left untreated lesions can ulcerate and without proper diagnosis and treatment can lead to significant morbidity and long term disability [4]. Despite the first identification of BU at the end of the 19^th^ century, we still do not have a complete understanding of the mode of transmission, and there is no vaccine [5]. The introduction of a fully oral treatment regimen by the WHO (utilizing clarithromycin and rifampicin) has revolutionized BU treatment [6]; however, there are still issues associated with the length of treatment (8 weeks), tolerability and access to the antibiotics, presenting opportunities to further improve this regimen.

*M*. *marinum* is genetically closely related to *M*. *ulcerans*, yet causes non-ulcerative, granulomatous skin lesions that are commonly associated with aquaria, giving these infections the common name of “fish tank granuloma” [7]. While mortality associated with *M*. *ulcerans* and *M*. *marinum* infections is low, this is not the case for infections with another NTM, *M*. *chimaera*. First identified in 2004, *M*. *chimaera* was responsible for a global outbreak linked to contaminated heater-cooler devices used in cardiac bypass surgery [8,9]. With a 50–60% mortality rate and prolonged treatment regimen of 12–18 months with an antibiotic cocktail of macrolides, ethambutol and rifamycins, new, better tolerated and more rapidly effective regimens are required [10].

Improvements to treatment regimens for NTM infections require the development of new antibiotics. SPR719 (formerly known as VXc-486) is a novel aminobenzimidazole that targets the essential ATPase activity of the GyrB subunit of DNA gyrase in mycobacteria [11,12]. Several studies have shown that SPR719 is active *in vitro* against *M*. *tuberculosis* and a range of NTMs, including *M*. *abscessus*, *M*. *avium* complex and *M*. *kansasii* [12,13]. Further work has shown that the oral prodrug form known as SPR720, is effective at controlling tuberculosis in mice and is also active in a mouse model of pulmonary *M*. *avium* infection [14–16]. Recently, SPR720 was granted investigational new drug (IND) status by the FDA as a novel oral agent for pulmonary NTM infections and has recently begun a Phase IIa clinical trial for this indication (https://clinicaltrials.gov/ct2/show/NCT04553406). Given the demonstrated efficacy against pulmonary NTMs, the purpose of this study was to explore the activity of SPR719 against skin and cardiac mycobacterial pathogens (*M*. *ulcerans*, *M*. *marinum*, *M*. *chimaera*) *in vitro*.

## Methods

### Isolates and growth conditions

*M*. *ulcerans* cultures were initially grown on Brown and Buckle agar slopes, while *M*. *marinum and M*. *chimaera* isolates were grown on 7H10 agar plates containing 10% OADC supplement (Difco). All *M*. *ulcerans* and *M*. *marinum* isolates were incubated at 30°C, while *M*. *chimaera* isolates were incubated at 37°C. The tested isolates and their origins are listed in Tables 1–3.

**Table 1:** *M*. *ulcerans* strains used in this study and their MICs.

			MIC (μg/mL)		
*M*. *ulcerans* strain	Country source	Source type	SPR719	Rifampicin	Clarithromycin
1615	Malaysia	Clinical	0.125	0.25	1
JKD8049	Australia	Clinical	0.125	0.25	1
Agy99	Ghana	Clinical	0.125	0.25	0.5
NM2310-1	Ghana	Clinical	0.125	2	2
IC21 Kouakou	Ivory Coast	Clinical	0.125	1	2
Lolo	Ivory Coast	Clinical	0.25	1	1
S81-9	Benin	Clinical	0.125	0.25	0.5
S53-9	Benin	Clinical	0.25	1	2
11–263	Cameroon	Clinical	0.125	0.25	1
11–394	Cameroon	Clinical	0.125	0.25	1
MIC range			**0.125–0.25**	**0.125–2**	**0.5–2**

**Table 2:** *M*. *marinum* strains used in this study and their MICs.

			MIC (μg/mL)		
*M*. *marinum* strain	Country source	Source type	SPR719	Rifampicin	Clarithromycin
M	Unknown	Human	0.5	0.5	0.5
1717	USA	Armadillo	1	1	2
1726	USA	Armadillo	0.5	0.5	0.5
KSW4	Unknown	Fish	1	1	1
KSW1	Unknown	Fish	1	1	1
**MIC range**			**0.5–1**	**0.5–1**	**0.5–2**

**Table 3:** *M*. *chimaera* strains used in this study, their MICs and a summary of genomic SNPs.

			MIC (μg/mL)			SNPs vs ANZ045*
*M*. *chimaera* strain	Country source	Source type	SPR719	Rifampicin	Clarithromycin	
DMG1600123	NZ	Heater-cooler unit	0.25	2	0.5	228
DMG1600132	Australia	Clinical	0.125	2	0.5	26
DMG1600133	Australia	Clinical	2	8	4	5082
DMG1600134	Australia	Heater-cooler unit	0.5	1	1	3415
DMG1700722	Australia	Clinical	<0.03	2	0.5	37,933
**MIC range**			<**0.03–2**		**0.5–4**	

* Shows number of SNPs in genome of each isolate when compared to the reference *M*. *chimaera* strain ANZ045.

### Preparation of antibiotic solutions

SPR719 was provided by Spero Therapeutics Ltd. Rifampicin and clarithromycin were purchased from Sigma-Aldrich. Stock solutions for all antibiotics were prepared in DMSO and were prepared freshly before dilution in agar plates. Antibiotics and plates containing antibiotics were protected from light during storage and incubation.

### Preparation of bacteria and determination of MIC

Currently, there are no CLSI prescribed methods for the susceptibility testing of *M*. *ulcerans*, *M*. *marinum* and *M*. *chimaera* and laboratories are recommended to develop their own testing methods [17]. As such, we used standardized media and techniques recommended by CLSI for MIC testing of the NTM species [17].

All *M*. *ulcerans* strains used were clinical isolates and were prepared for MIC determination from colonies grown on Brown and Buckle agar. Colonies were scraped, suspended in distilled water and diluted to a McFarland standard equivalent of 0.1. 100ul of this bacterial suspension was inoculated onto BD Middlebrook 7H10 agar (Becton Dickinson) plates supplemented with 10% BBL Middlebrook OADC enrichment (Becton Dickinson) and containing twofold dilutions of SPR719 (32 to 0.03 μg/ml), rifampicin (8 to 0.25 μg/ml) or clarithromycin (2 to 0.25 μg/ml). *M*. *ulcerans* containing plates were incubated for 10 weeks at which point they were examined for bacterial growth. Plates without antibiotics were used as controls. The MIC was defined as the lowest drug concentration to inhibit growth of at least 99% of CFU on control plates.

For *M*. *marinum* and *M*. *chimaera* cells were scraped from 7H10 agar plates and suspended in distilled water to a McFarland standard equivalent of 0.1. 100ul of this bacterial suspension was inoculated on duplicate 7H10 agar plates with increasing concentrations of twofold dilutions of SPR719 (32 to 0.03 μg/ml), rifampicin (8 to 0.25 μg/ml) or clarithromycin (2 to 0.25 μg/ml). Plates were incubated for 2 weeks for *M*. *marinum* and 3 weeks for *M*. *chimaera*. MICs were determined as the lowest concentration of antibiotic that did not yield bacterial growth. Plates containing solvent only were used as controls.

### Genomic analysis of *M*. *chimaera* isolates

Genome sequences for the five *M*. *chimaera* isolates investigated in this study were analysed to determine the number of SNPs per genome relative to the reference strain ANZ045 (NCBI accession NZ_LT703505.1). Genomes were sequenced as part of a previous study (data accessible at NCBI PRJEB15375) [18]. SNP analysis was performed using Snippy 4.6.0 (https://github.com/tseemann/snippy).

### Analysis of *gyrB* loci from *M*. *ulcerans*, *M*. *marinum* and *M*. *chimaera*

The nucleotide sequence of *gyrB* was extracted from the genome sequences of the *M*. *ulcerans* and *M*. *chimaera* strains utilized in this study, as well as *M*. *marinum* M, *M*. *tuberculosis* H37Rv and *M*. *abscessus* ATCC 19977. Gene sequences were translated to amino acid sequences and aligned using CLUSTALW, as implemented in Geneious R9.1.8 (Biomatters Ltd).

## Results

### SPR719 inhibition of *M*. *ulcerans* growth

Ten different *M*. *ulcerans* clinical isolates were tested to determine the *in vitro* activity of SPR719. SPR719 was found to be active against all *M*. *ulcerans* isolates tested with a MIC range of 0.125–0.25 μg/ml (Table 1). These results are comparable to, and for some strains, better than the comparator antibiotics rifampicin and clarithromycin. These data show that *M*. *ulcerans* is susceptible to SPR719. Growth of all *M*. *ulcerans* strains was observed on control plates containing solvent only.

### SPR719 activity against *M*. *marinum* and *M*. *chimaera*

*M*. *marinum* and *M*. *chimaera* were also tested for their susceptibility to SPR719 by plating the bacteria onto plates containing increasing concentrations of SPR719. All *M*. *marinum* strains were also susceptible to SPR719 (Table 2), although less so than *M*. *ulcerans*. The tested *M*. *marinum* strains had a MIC range of 0.5–1 μg/ml, which was similar to the MIC range for the comparator compounds rifampicin and clarithromycin (Table 2). *M*. *chimaera* was also susceptible to SPR719, however isolates were more varied in their responses with a MIC range of <0.03–2 μg/ml (Table 3). Interestingly, one isolate (DMG1600133) was less susceptible to both SPR719 and clarithromycin than the other tested isolates, while isolate DMG1700722 appeared to be highly susceptible to SPR719, with the lowest MIC (<0.03 μg/ml) of all mycobacteria tested in this study.

To further investigate the variable responses of the *M*. *chimaera* isolates to SPR719, we looked for genetic divergence of these isolates. The genomes of the *M*. *chimeara* isolates used in this study were previously sequenced as part of an outbreak investigation [18]. SNP analysis of the tested *M*. *chimaera* isolates (Table 3) showed DMG1600132 (SPR719 MIC 0.125 μg/ml) had only 26 SNPs when compared to the ANZ045 reference genome, which represents a clone linked to the global heater-cooler outbreak [19]. This means that SPR719 has useful *in vitro* activity against the lineage of *M*. *chimaera* linked to prosthetic heart valve infections. The other two *M*. *chimaera* isolates that are more divergent from the outbreak clonal group (DMG1600133 with >5000 SNPs and DMG1700722 with >37,000 SNPs) help explain the variation in MICs observed. None of the mutations identified in either DMG1600133 or DMG1700722 were in genes previously associated with reduced susceptibility to SPR719 (e.g. *gyrB*), suggesting that there may be other intrinsic factors related to the different susceptibilities to SPR719 within these bacteria.

### GyrB mutations associated with SPR719 non-suceptibility

To further investigate the activity of SPR719 on the tested *M*. *ulcerans*, *M*. *marinum* and *M*. *chimaera* isolates, we looked at GyrB residues that have previously been associated with decreased SPR719 susceptibility. In *M*. *tuberculosis* GyrB these residues are A92 and S208 (*M*. *tuberculosis* GyrB numbering), where the A92S and S208A mutations resulted in decreased susceptibility to SPR719 and other related GyrB targeting antibiotics [12,20,21]. Investigation of the equivalent A92 site in the *M*. *ulcerans* and *M*. *chimaera* strains tested here, as well as the *M*. *marinum* ‘M’ strain genome, revealed that these organisms all possess serine at position 92 and threonine at position 208 (Table 4), yet remain susceptible to SPR719.

**Table 4:** Amino acid residues at GyrB positions associated with SPR719 non-susceptibility.

	GyrB position*	
Strain**	92	208
*M*. *tuberculosis* H37Rv	Ala	Ser
*M*. *abscessus* ATCC 19977	Ser	Thr
*M*. *ulcerans* Agy99	Ser	Thr
*M*. *marinum* ‘M’	Ser	Thr
*M*. *chimaera* DMG1600132	Ser	Thr

* Numbers refer to positions in *M*. *tuberculosis* GyrB.

** Although only one isolate each of *M*. *ulcerans* and *M*. *chimaera* is listed in the table, all *M*. *ulcerans* and *M*. *chimaera* isolates used in this study had identical amino acid residues at the positions noted. The genomes of the other *M*. *marinum* isolates used in this study were unavailable for study.

## Discussion

SPR719 is a novel aminobenzimidazole (previously known as VXc-486) that targets DNA gyrase subunit B (GyrB) [11,22,23]. While GyrA is the target of the fluoroquinolone class of antibiotics, several of which are active against mycobacteria [24–26], there have been relatively few identified compounds that target GyrB. The essential nature of the DNA gyrase complex (GyrA and GyrB) for bacterial DNA replication combined with the lack of a human orthologue, make this an appealing anti-bacterial target. Interestingly, in contrast to other bacterial species that have gyrase and topoisomerase IV subunits (ParC and ParE), genomic studies have shown that mycobacteria lack topoisomerase IV making DNA gyrase the sole complex with topoisomerase activity in this group of organisms [27–29].

Previous studies have shown that SPR719 is active against *M*. *tuberculosis* both *in vitro* and in a mouse model of infection, including extensively drug-resistant strains [12]. Furthermore, SPR719 was shown to be active against several non-tuberculosis mycobacteria, including *M*. *abscessus*, *M*. *avium*, *M*. *chelonae*, *M*. *kansasii* and *M*. *marinum* [12,13]. A recent Phase I clinical trial has also shown SPR720 (the oral prodrug of SPR719) to be well tolerated at doses necessary to give plasma drug concentrations in the range required for *in vivo* activity against non-tuberculosis mycobacteria [27]. Here, we have shown that the SPR719 activity spectrum extends to other non-tuberculosis mycobacteria, including *M*. *ulcerans*, the causative agent of the neglected tropical disease, Buruli ulcer.

The MIC range of SPR719 against *M*. *ulcerans* (0.125–0.25 μg/ml) is similar to or better than that for other non-tuberculosis mycobacteria (MIC 0.23 μg/ml against *M*. *avium* [12], MIC range 0.12–8 μg/ml against a range of *M*. *abscessus* subspecies [13]). However, *M*. *ulcerans* appears to be less susceptible to SPR719 than *M*. *tuberculosis* or *M*. *kansasii* (MIC range 0.008–0.125 μg/ml and 0.06–2 μg/ml, respectively) [12]. Our results for *M*. *marinum* MICs are in accordance with those published previously (MIC range 0.12–1 μg/ml) and confirm that *M*. *marinum* is also susceptible to SPR719 [13]. Despite the high level of genetic similarity between *M*. *ulcerans* and *M*. *marinum* (99%), the discrepancy in MICs may be due to higher number of pseudogenised transporters and efflux systems in *M*. *ulcerans* compared to *M*. *marinum* [30,31], although other variables, such as different growth rates between the organisms, may also play a role. The MIC range for *M*. *chimaera* (<0.03–2 μg/mL) was wider than for the other two pathogens tested here, however it is not substantially different from that seen previously for other NTMs such as *M*. *abscessus* [12]. Furthermore, such variability has been seen across a much larger range of *M*. *chimaera* isolates for antibiotics such as amikacin and linezolid [32].

Furthermore, the genetic diversity of *M*. *chimaera* isolates tested was substantial, helping to explain the variation in MICs observed. Significantly however, SPR719 is active against the *M*. *chimaera* lineage associated with infections caused by contaminated heater-cooler units used in cardiac surgery.

Previous studies have shown that A92S and S208A mutations in *M*. *tuberculosis* GyrB resulted in decreased susceptibility to SPR719 and other related antibiotics that target GyrB [12,20,21]. However, as has also been noted previously, *M*. *abscessus* contains a natural serine at position 92, yet is still susceptible to SPR719 [12]. All organisms investigated in this study also possess a serine at position 92 and additionally a threonine at position 208, which is different from that seen in *M*. *tuberculosis* (Table 4). As serine and threonine are both hydroxylic amino acids and differ minimally, it seems logical that this change at position 208 is insufficient to cause decreased SPR719 susceptibility in these bacteria. Yet, the fact that S92 is common to multiple organisms that are still susceptible to SPR719, yet causes resistance in *M*. *tuberculosis*, suggests that these may not be the only residues, or factors, important in reduced susceptibility to SPR719 in these organisms.

Several recent studies have sought to find better tolerated and more effective drug combinations than those currently used for the treatment of NTM infections caused by the organisms under study here. For example, higher doses of rifamycins alone or in combination with clofazimine have been shown to shorten treatment time of Buruli ulcer infections in mice [33,34], while other studies have shown multi-drug regimens containing telacebec (also known as Q203) can sterilize murine *M*. *ulcerans* infections in as little as 14 days [35,36] and even a single dose of Q203 was found to sterilize mouse lesions [37]. Given that SPR720 was found to synergize with both clarithromycin and ethambutol in a *M*. *avium* chronic infection model to significantly reduce bacterial burden [15], our results warrant further investigation of the ability of SPR720 to enhance treatment regimens for the NTM investigated in this study.

The novel aminobenzimidazole, SPR719 inhibited the growth of the neglected mycobacterial pathogens *M*. *ulcerans*, *M*. *marinum* and *M*. *chimaera* with a MIC range of 0.5–4 μg/ml across the three species. These data highlight the efficacy of SPR719 against non-tuberculosis mycobacteria and, combined with recent data demonstrating *in vivo* efficacy against *M*. *avium* and the recently completed Phase 1 safety assessment in humans, indicates the potential of SPR719/SPR720 to be investigated for the treatment of infections due to NTM, including *M*. *ulcerans*, *M*. *marinum* and *M*. *chimaera*.
